# Supplementary material for: Chronic emotional stress and mediating role of Interleukin-6 in the association with cardiometabolic disorders in a multiethnic middle-aged and older US population
Source: Int J Cardiol Cardiovasc Risk Prev. 2025 Sep 6;27:200510. doi: 10.1016/j.ijcrp.2025.200510 (PMC12475582; doi:10.1016/j.ijcrp.2025.200510)
Supplement: Multimedia component 1 [file mmc1.docx]

**Supplementary Table 1:** Chronic Stress Questionnaire

|  | No | Yes | Not very stressful | Moderately stressful | Very stressful |
| --- | --- | --- | --- | --- | --- |
| **Chronic Stress 1: Have you had a serious ongoing health problem?** | 0 | 1 |  |  |  |
| Chronic Stress 1a: Has this been a problem for six months or more? | 0 | 1 |  |  |  |
| Chronic Stress 1b: Would you say this problem has been? |  |  | 1 | 2 | 3 |
| **Chronic Stress 2: Has someone close to you had a serious ongoing health problem?** | 0 | 1 |  |  |  |
| Chronic Stress 2a: Has this been a problem for six months or more? | 0 | 1 |  |  |  |
| Chronic Stress 2b: Would you say this problem has been? |  |  | 1 | 2 | 3 |
| **Chronic Stress 3: Have you had ongoing difficulties with your job or ability to work?** | 0 | 1 |  |  |  |
| Chronic Stress 3a: Has this been a problem for six months or more? | 0 | 1 |  |  |  |
| Chronic Stress 3b: Would you say this problem has been? |  |  | 1 | 2 | 3 |
| **Chronic Stress 4: Have you experienced ongoing financial strain?** | 0 | 1 |  |  |  |
| Chronic Stress 4a: Has this been a problem for six months or more? | 0 | 1 |  |  |  |
| Chronic Stress 4b: Would you say this problem has been? |  |  | 1 | 2 | 3 |
| **Chronic Stress 5: Have you had ongoing difficulties in a relationship with someone close to you?** | 0 | 1 |  |  |  |
| Chronic Stress 5a: Has this been a problem for six months or more? | 0 | 1 |  |  |  |
| Chronic Stress 5b: Would you say this problem has been? |  |  | 1 | 2 | 3 |
| **Chronic Stress 6: Has someone close to you had an ongoing problem with alcohol or drug use?** | 0 | 1 |  |  |  |
| Chronic Stress 6a: Has this been a problem for six months or more? | 0 | 1 |  |  |  |
| Chronic Stress 6b: Would you say this problem has been? |  |  | 1 | 2 | 3 |
| **Chronic Stress 7: Have you been helping someone close to you, who is sick, limited or frail?** | 0 | 1 |  |  |  |
| Chronic Stress 7a: Has this been a problem for six months or more? | 0 | 1 |  |  |  |
| Chronic Stress 7b: Would you say this problem has been? |  |  | 1 | 2 | 3 |
| **Chronic Stress 8: Have you had another ongoing problem not listed here?** | 0 | 1 |  |  |  |
| Chronic Stress 8a: If yes, please describe: |  |  |  |  |  |
| Chronic Stress 8b: Has this been a problem for six months or more? | 0 | 1 |  |  |  |
| Chronic Stress 8c: Would you say this problem has been? |  |  | 1 | 2 | 3 |
| **Total Chronic Stress score** |  |  |  |  |  |
